# The Performance and Spatial Distribution of Membrane Fouling in a Sequencing Batch Ceramic Membrane Bioreactor: A Pilot Study for Swine Wastewater Treatment

**DOI:** 10.3390/membranes14060142

**Published:** 2024-06-18

**Authors:** Wenhui Yue, Yanlin Chen, Qianwen Sui, Libing Zheng, Tharindu Ritigala, Yuansong Wei

**Affiliations:** 1 State Key Joint Laboratory of Environment Simulation and Pollution Control, Research Center for Eco-Environmental Sciences, Chinese Academy of Sciences, Beijing 100085, China; yuewenhui17@mails.ucas.ac.cn (W.Y.); chen_yanlin@ctg.com.cn (Y.C.); qwsui@rcees.ac.cn (Q.S.); lbzheng@rcees.ac.cn (L.Z.); tharindu@bewg.net.cn (T.R.); 2 Laboratory of Water Pollution Control Technology, Research Center for Eco-Environmental Sciences, Chinese Academy of Sciences, Beijing 100085, China; 3 University of Chinese Academy of Sciences, Beijing 100049, China

**Keywords:** ScMBR, swine wastewater, membrane fouling, spatial distribution

## Abstract

The extensive application of ceramic membranes in wastewater treatment draws increasing attention due to their ultra-long service life. A cost-effective treatment for high-strength swine wastewater is an urgent and current need that is a worldwide challenge. A pilot-scale sequencing batch flat-sheet ceramic membrane bioreactor (ScMBR) coupled with a short-cut biological nitrogen removal (SBNR) process was developed to treat high-strength swine wastewater. The ScMBR achieved stable and excellent removal of COD (95.3%), NH_4_^+^-N (98.3%), and TN (92.7%), though temperature went down from 20 °C, to 15 °C, to 10 °C stepwise along three operational phases. The COD and NH_4_^+^-N concentrations in the effluent met with the discharge standards (GB18596-2001). Microbial community diversity was high, and the genera *Pseudomonas* and *Comamonas* were dominant in denitritation, and *Nitrosomonas* was dominant in nitritation. Ceramic membrane modules of this pilot-scale reactor were separated into six layers (A, B, C, D, E, F) from top to bottom. The total filtration resistance of both the top and bottom membrane modules was relatively low, and the resistance of the middle ones was high. These results indicate that the spatial distribution of the membrane fouling degree was different, related to different aeration scour intensities demonstrated by computational fluid dynamics (CFD). The results prove that the membrane fouling mechanism can be attributed to the cake layer formation of the middle modules and pore blocking of the top and bottom modules, which mainly consist of protein and carbohydrates. Therefore, different cleaning measures should be adopted for membrane modules in different positions. In this study, the efficient treatment of swine wastewater shows that the ScMBR system could be applied to high-strength wastewater. Furthermore, the spatial distribution characteristics of membrane fouling contribute to cleaning strategy formulation for further full-scale MBR applications.

## 1. Introduction

The exponential world population growth has enhanced meat consumption worldwide [1], and China accounts for 48.4% of worldwide pork production [2]. With the expansion of concentrated and industrialized pork production, the quantities of swine wastewater (SW) generated during the pig farming process has increased rapidly [3,4], to approximately 0.16 billion tons per year in China [5]. According to the Second National Survey of Pollution Sources of China in 2017, the animal industry was one of the main sources of water pollutants, and the swine industry accounted for 60% [6]. Discharge of SW without effective treatment could lead to the eutrophication of its high contents of organic matter and nutrients, including nitrogen and phosphorus [7]. Sufficient and eco-friendly treatments are required [8].

Short-cut biological nitrogen removal (SBNR) based on nitritation and denitritation processes saves aeration and carbon sources and reduces sludge production compared with traditional nitrification and denitrification processes [9]. Considering the high COD and ammonia concentrations and the relatively low C/N ratio of swine wastewater, the SBNR process is suitable to apply for simultaneous COD and TN removal. Multiple reactor configurations were investigated for efficient swine wastewater treatment, such as a sequencing batch reactor (SBR) [10], an aerobic granular sludge batch reactor (GSBR) [11], an integrated fixed-biofilm activated sludge sequencing batch reactor (IFAS-SBR) [12], and an up-flow anaerobic sludge bed (UASB) [13]. The SBR was considered and suggested as the best approach to treat SW for its even mix of good mass transfer and simple operation [14]. However, bad settlement, sludge washout, and bad effluent quality restricted SBR spreading and application. The membrane bioreactor (MBR) demonstrated advantages in solid–liquid separation and efficient sludge retention, widely adopted by microfiltration or ultrafiltration [15]. Incorporating SBR and MBR, the sequencing batch membrane bioreactor (SMBR) could present a promising direction for low turbidity and good effluent quality in high-strength wastewater treatment, such as dye-containing wastewater [16], acrylic fiber wastewater [17], and food waste digestate [18] treatment. Sui et al. focused on a real-time automatic control strategy for the combination of a short-cut biological nitrogen removal (SBNR) process and an SMBR device, and achieved an HRT of 6.0 d and a TN loading rate of 0.02 kgN/(kgVSS d) [9]. Tharindu et al. used SMBR in both SW and food waste digestate treatment, achieving stable and excellent removal of COD (96.05 ± 0.2%, 97.39 ± 0.2%) and TN (97.30 ± 0.3%, 97.44 ± 0.3%), respectively [18]. However, these studies were conducted in lab-scale systems, pilot-scale research is necessary for the full-scale application of SMBR in SW treatment.

In addition, ceramic membrane-based treatments have already gained considerable attention in recent decades [19], being applied in various aspects, i.e., oily wastewater [20], desalination, and municipal wastewater [21], though most membrane applications adopt organic membranes for a lower capital cost. The cost of ceramic membranes is comparable to their polymeric counterparts, due to their significant advantages like higher structural stability and a longer lifespan, according to techno-economic models [22] and life cycle assessments [23]. Shi et al. reported the feasibility of using CMBR to treat coal chemical wastewater with toxic and refractory pollutants [24]. Sun et al. applied the combined SBR and Ceramic MBR (CMBR) to high-salinity oil-bearing wastewater treatment, which could save 62.5% of the energy cost compared to the conventional MBR process [25]. Ninomiya et al. achieved a higher-flux operation of CMBR using an intensive cleaning strategy combining mechanical scouring with granules and chemically enhanced backwashing, which was possible with ceramic flat-sheet membranes instead of organic membranes [26]. Though the efficacy of ceramic membrane bioreactors (CMBRs) has been demonstrated for various wastewater treatments, membrane fouling remains one of the bottlenecks in the widespread application of MBRs as well as CMBRs. Compared to polymeric membranes, ceramic membranes are less prone to organic fouling due to their higher hydrophilicity and lower surface charge. As for biofouling, potentially the most harmful problem for membranes and the most challenging to mitigate, the build-up of it is closely linked to the biomass characteristics and concentrations of extracellular polymeric substances (EPS). In the context of ceramic membranes, as compared to organic fouling, information on the characteristics of biofouling and its mitigation is scarce [19].

Therefore, the main objectives of this study are (1) to explore the feasibility and efficiency of removing organic matter and nitrogen from swine wastewater using a pilot-scale sequencing ceramic membrane bioreactor (ScMBR); (2) to investigate the shifts in the microbial community of bulk sludge; (3) to explore the overall spatial distribution of membrane fouling of ScMBR, and specifically, the organic and biological fouling characteristics.

## 2. Material and Methods

### 2.1. Wastewater and Seed Sludge Characteristics

The influent swine wastewater was continuously supplied from a piggery located in the suburban area of Beijing, China, with 20,000 head in stock. It was pretreated by preformed precipitation and subsequent solid–liquid separation by spiral extrusion, and then transported into the pilot-scale reactor. Seed sludge was taken from an aerobic tank of a full-scale A^2^/O wastewater treatment plant and the initial MLSS inside of the reactor was around 6000 mg/L, MLVSS was about 4500 mg/L.

### 2.2. Reactor Setup and Operation

**Figure 1:**
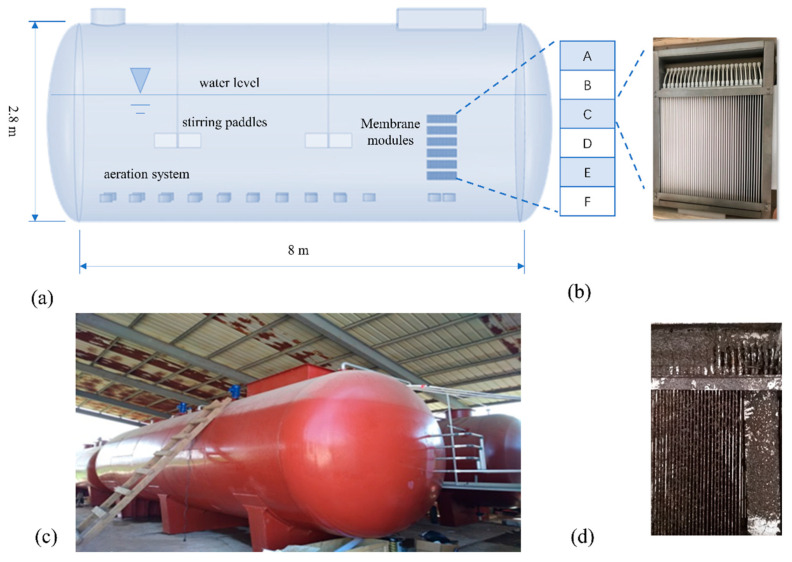
Schematic diagram of ScMBR (**a**); membrane module arrangement (**b**); on-site photo of ScMBR (**c**); fouled membrane modules (**d**).

The pilot-scale sequencing batch ceramic membrane bioreactor (ScMBR) (shown in Figure 1) was re-designed and manufactured based on a successful lab-scale one. It was a horizontal cylinder (D = 2.8 m, L = 8 m) made of carbon steel (d = 8 mm), with the total volume of 50 m^3^ and the working volume of 30~35 m^3^ (Chuangdi, Beijing, China). Two stirring paddles were equipped for even mixing and aeration system was installed at the bottom of the reactor for necessary oxygen supply.

Membrane modules were divided into 6 layers (A/B/C/D/E/F) from top to bottom, and placed to the side of the ScMBR’s interior. Every layer consisted of 48 pieces of flat ceramic membrane (0.1 µm, Boxing, Jiangxi, China) with a surface area of 0.055 m^2^ on each side. In total, there were 288 pieces of membrane with an effective filtration surface area of 31.6 m^2^. The interval between each piece was 0.8 m. Every piece of membrane was set vertically to the air flushing system, beneath the membrane module. An outlet pipe was equipped to collect effluent in each layer, then they were assembled and connected to a suction pump. When the pressure of the suction pump—the transmembrane pressure (TMP)—exceeded 30 kPa, the membrane module was backwashed by tap water, sodium hypochlorite solution (2500 mg/L), and hydrochloric acid (pH = 2) step by step for filtration flux recovery.

The designed treatment capacity was 10 m^3^/d and the actual capacity was 5~6 m^3^/d. The hydraulic retention (HRT) was controlled as 5.6~6.6 d, sludge retention time (SRT) was 15~20 d. The operational pattern of ScMBR included 0.5 h feeding, 1.5 h anoxic stirring, 3 h oxic phase (DO = 0.5~2 mg/L), and 0.6~1 h discharging based on membrane modules in each cycle.

### 2.3. Membrane Resistance Analysis

TMP and flux values were recorded during every step of the backwashing process and were used for calculating filtration resistance [27]. According to the resistance-in-series model, and taking backwashing for flux recovery into account, irreversible fouling (*R_i_*), pore blocking resistance (*R_p_*), and cake layer resistance (*R_c_*) contribute to total resistance (*R_t_*). The reversible fouling was removed with hydraulic backwashing and chemical backwashing. And *R_i_* includes membrane resistance (*R_m_*). Therefore, resistance values were computed using Equations (1)–(4).

(1) $$J_{f}=\frac{TMP_{f}}{\mu R_{t}}$$

(2) $$J_{w}=\frac{TMP_{w}}{\mu (R_{t}-R_{c})}$$

(3) $$J_{ch}=\frac{TMP_{ch}}{\mu (R_{t}-R_{c}-R_{p})}$$

(4) $$R_{t}=R_{i}+R_{p}+R_{c}$$

where *J* is the flux (Lm^−2^h^−1^), *TMP* is the transmembrane pressure (Pa), *J_f_* and *TMP_f_* refer to final flux and pressure (without washing), *J_w_* and *TMP_w_* refer to flux and pressure after washing with clean water, *J_ch_* and *TMP_ch_* refer to flux and pressure after washing with chemicals, *μ* is the viscosity of permeate (Pa·s), and *R* is the filtration resistance (m^−1^). Also, resistance distribution was calculated with the following equations.

(5) $$R_{c}\left(\%\right)=\frac{R_{c}}{R_{t}}\times 100$$

(6) $$R_{p}\left(\%\right)=\frac{R_{p}}{R_{t}}\times 100$$

(7) $$R_{i}\left(\%\right)=\frac{R_{i}}{R_{t}}\times 100$$

### 2.4. Flow Field Distribution

In the ScMBR membrane module, the degree of membrane fouling varied with the high and low locations during the swine wastewater treatment. The different flow velocity caused by the air flushing system was believed to be the main factor; therefore, a three-dimensional single-phase computational fluid dynamics (CFD) approach was used to evaluate the flow regime in the ScMBR reactor, especially the membrane module region. The geometry of the structure was designed using SpaceClaim 17.0 (ANSYS, Waltham, MA, USA); structured hexagonal grids were employed for their superior mesh quality and calculation of convergence control. The details about postprocessing and the simulation process can be found in the Supporting Information.

### 2.5. Analytical Methods

#### 2.5.1. Sampling

Samples of influent and effluent were collected daily to estimate the nitrogen removal performance of the ScMBR reactor. Mixed liquid samples were taken monthly to investigate the microbial community structure.

Samples of fouled membrane, foulant attached to the membrane surface, and soak solution for membrane cleaning were taken in order to study the fouling of different membrane modules placed in different locations.

#### 2.5.2. Measurement Methods

The concentrations of physicochemical parameters, such as ammonia, nitrite, nitrate, MLSS, and MLVSS were performed according to the standard methods. COD was measured by photometric measurements (DR 4000, Hach, Loveland, CO, USA). DNA was extracted using the FAST DNA Extraction Kit (MP biomedicals, Santa Ana, CA USA) and the microbial community structure was analyzed using high-throughput sequencing according to the methods from previous studies [9].

The morphology of the membranes (clean, fouled, and stripped) was observed using a field emission scanning electron microscope (FE-SEM, HITACHI SU8020, Hitachi, Tokyo, Japan). The samples were freeze-fractured with liquid nitrogen for cross-sectional observations, and samples were coated with gold nanoparticles under a vacuum with a HITACHI E-1010 Ion device (Hitachi, Japan) prior to SEM observations.

The EPS of cake layer samples was extracted using a modified heat method according to Li [28]. The contents of proteins and carbohydrates in EPS were determined according to the methods developed by Lowry and Dubois, respectively.

As for the soak solution produced by membrane chemical cleaning, TOC and TIC were measured by a TOC analyzer (Vario TOC, Elementar, Langenselbold, Germany). The molecule weight distribution was analyzed by high-performance size exclusion chromatography (HPSEC, Waters 1525, Waters, Milford, MA, USA). Three-dimensional excitation–emission matrix fluorescence spectra (3DEEM) were used to characterize the DOM through a fluorescence spectrophotometer (F-7000, Hitachi, Tokyo, Japan).

#### 2.5.3. Statistical Analysis

Statistical calculations and data analysis were performed using the SPSS 20 statistical software package (IBM, Armonk, NY, USA), and figures were plotted with OriginPro 2018 (OriginLab, Northampton, MA, USA). Spearman rank correlations were used to assess the associations between operating variables and microbial parameters, together with biochemical parameters, and a *p* value < 0.05 was considered statistically significant. Heatmaps were plotted through the HemI 1.0 (CUCKOO, Wuhan, China).

## 3. Results and Discussion

### 3.1. Pollutant Removal Performance

**Figure 2:**
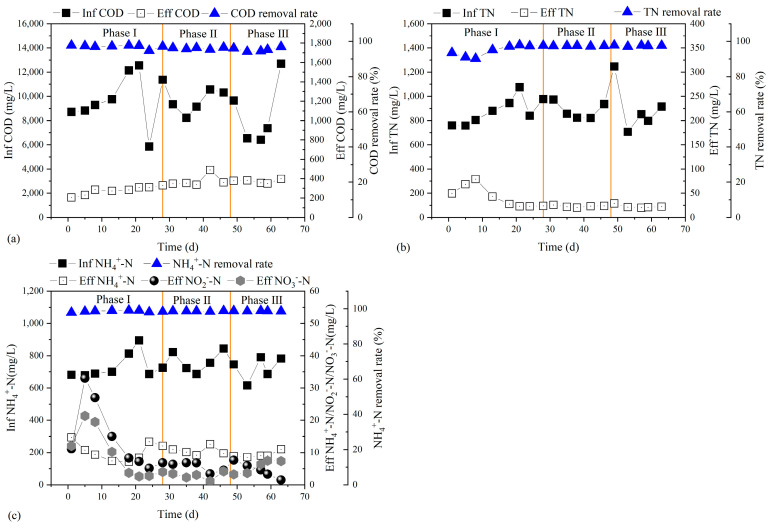
Operational performance of ScMBR for COD removal (**a**), TN removal (**b**), and ammonia removal (**c**).

Though the influent of high-strength swine wastewater fluctuated a lot, the ScMBR still performed excellently in the removal of COD, TN, and NH_4_^+^-N, as shown in Figure 2. The effluent quality including COD, NH_4_^+^-N, and SS met the Discharge Standard of Pollutants for Livestock (GB18596-2001).

Throughout the whole operational period of 63 days, the influent concentration of COD was around 9377 mg/L and the effluent concentration of COD was around 332 mg/L. The COD removal efficiency was rather robust, with an average removal rate of 96% even with fluctuating feeding and organic loading rates due to the application of membrane separation. The removal of COD in this study was higher than in other studies, like those on the conventional SBR or A/O systems [29]. Specifically, the effluent of COD showed an increasing trend from 279 mg/L to 378 mg/L and 373 mg/L in Phase I, Phase II, and Phase III, respectively. It could be caused by the decrease in microbial activity for organic matter decomposition due to the ambient temperature drop.

As for the nitrogen removal performance, the influent of TN was 880 mg/L in Phase I and the effluent of TN increased to a peak value of 79 mg/L on Day 8, with a TN removal efficiency of 89%. After the start-up period, the effluent of TN decreased to 29 mg/L. At the same time, TN removal efficiency increased to 97%. As for ammonia, the average influent concentration was 740 mg/L and the average effluent concentration was only 10 mg/L. The average ammonia removal efficiency reached 98.6%. Compared with COD reduction, it was less affected by ambient temperature. Effluent nitrate concentration was higher than influent nitrate concentration during the start-up stage due to the poor nitrogen removal effect. In addition, the inoculation sludge taken from aerobic pool of WWTP carried a huge amount of nitrate. Along with stable operation, effluent nitrate concentration gradually decreased to 6 mg/L. The effluent nitrite concentration also decreased. The maximum specific growth rate of AOB is lower than NOB when the ambient temperature is below 20 °C, leading to low nitrite accumulation. The nitrite accumulation rate was 62% and 67% in Phase I and Phase II, respectively, and it was only 44% when the ambient temperature was 10 °C in Phase III.

### 3.2. Shifts in Microbial Community

Microbial community dynamics during long-term nitrogen removal were investigated at the phylum and genus levels by high-throughput sequencing technology, and the good coverage (>98.9%) of all samples suggested that a satisfactory sequencing depth could cover the whole microbial community. At the phylum level, *Proteobacteria* (43.29~47.72%), *Firmicutes* (16.67~23.02%), *Bacteroidetes* (9.58~16.32%), and *Chloroflexi* (6.53~9.06%) were dominant in all phases, accounting for more than 75% of the total abundance. *Proteobacteria* increased slightly, while *Bacteroidetes* and *Chloroflexi* decreased with temperature drops. The abundance of Actinobacteria increased gradually. The excessive proliferation of *Actinobacteria* could cause large amounts of foam and sludge bulking in wastewater treatment plants [30]. However, though *Actinobacteria* increased, sludge loss led by sludge bulking will not occur in this reactor due to the installation of the membrane modules.

At the genus level, *Nitrosomonas*, as a typical ammonia-oxidizing bacteria (AOB), was the dominant nitrifier in ScMBR. With good control of DO, the abundance of nitrite-oxidizing bacteria (NOB) including *Nitrospira*, *Nitrobacter*, and *Nitrolancea* was much lower than AOB, contributing to the accomplishment of short-cut nitrification and nitrite accumulation. *Candidatus Brocadia* was found in a full-scale swine wastewater treatment plant, whereas no members of the anammox genus were detected in this ScMBR, possibly due to the long-term operation under 15 °C. High diversity of denitrifying populations was detected. And the abundance of them was also high compared with the conventional A^2^O system, conducing to efficient nitrogen removal performance. *Pseudomonas*, *Comamonas*, *Thauera*, *Hyphomicrobium*, *Thermomonas*, *Paracoccus*, *Flavobacterium*, *Azoarcus*, *Thiobacillus*, and *Ralstonia* composed the denitrifying community. *Pseudomonas* is frequently discovered in high-strength nitrogen removal systems like swine wastewater [9] and landfill leachate [31]. It is reported as a kind of aerobic denitrifier and exists under relatively high DO conditions. With decreasing temperatures, the abundance of *Pseudomonas* went up, proving good adaptability to low temperatures. The abundance of *Comamonas* was also on the rise. *Comamonas* was detected in a simultaneous anammox and denitrification (SAD) system under an undulating seasonal temperature [32]. Themomonas and Thauera were reported to readily reduce nitrate and be responsible for nitrite accumulation [33]. The abundance of Thauera was well-maintained at around 15 °C, but dropped in Phase III to around 10 °C. Collectively, the changes in temperature were considered as the main factor trigging the bacterial shift.

### 3.3. Membrane Fouling Overview

**Figure 3:**
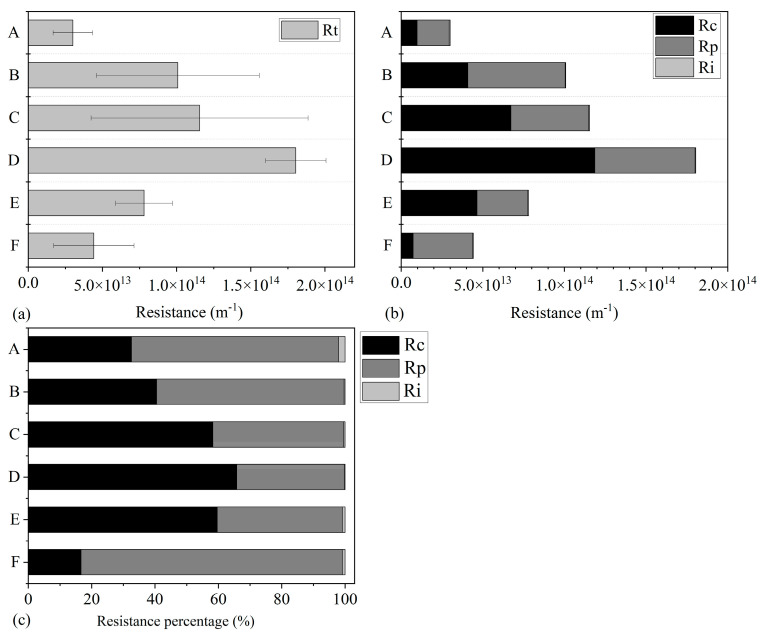
Total resistance distributions of different membrane modules (**a**); resistance distributions of different membrane modules (**b**); resistance proportion distributions of different membrane modules (**c**).

The total resistance (*R_t_*) of membrane units at different positions was tested at the end of the operational period to present a general profile of the different degree of membrane fouling, as shown in Figure 3. From top to bottom, *R_t_* increased first, reached a maximum at the D-Unit in the middle position, then dropped again. These observations indicate that the membrane modules were heavily fouled in the middle position and relatively less so at the top and bottom.

Total resistance was composed of cake layer resistance (*R_c_*), pore blocking resistance (*R_p_*), and irreversible resistance (*R_i_*). The spatial distributions and proportions are shown in Figure 3. As for the middle module assembly (C, D, E), cake layer resistance was the main component, occupying 58.22%, 65.79%, and 59.59% of the total resistance, respectively. This could be linked to the high cake layer (biofilm) formation and EPS production in the biofilm and the high PN/PS ratio of the middle assembly [34]. In contrast, *R_p_* was higher in both the top and bottom units than *R_c_*. However, regardless of the location of the membrane units, *R_i_* took a very small proportion of total resistance (0.2~2.03%), plausibly due to less inorganic compounds binding the functional groups on the membrane surface [35]. It verified the ultra-long service life of ceramic membranes due to their high structural and chemical stabilities and their anti-fouling properties [26]. Based on the above results, for middle units, the intensity and frequency of hydraulic backwashing should be enhanced to control cake layer resistance. In addition, the chemical cleaning process should be intensified for top module units.

**Figure 4:**
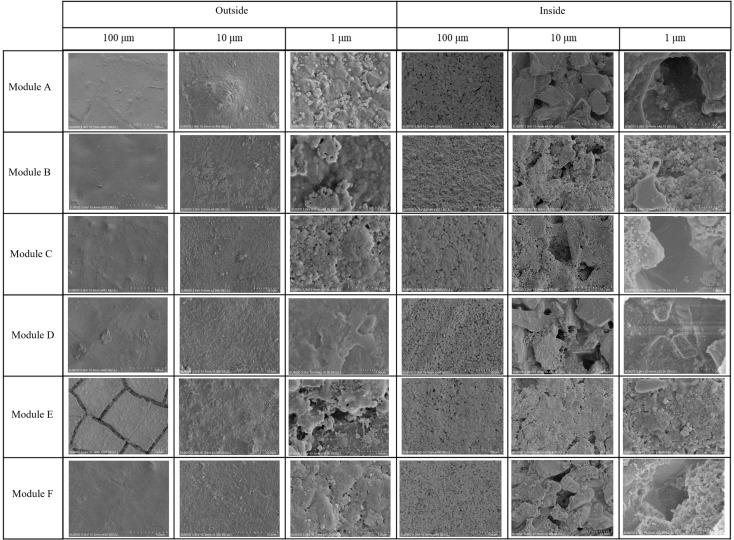
SEM images of the outsides and insides of membrane sheets from different modules at different magnifications.

In terms of membrane morphology, membrane autopsy analyses with SEM are widely used to determine the nature and formation of foulants on the membrane surface [36]. Figure 4 shows fouled membrane from different units under different magnifications, with both the outside surface and the inside surface being displayed. Compared with the inside surface, cake layer was thicker and denser on the outside surface no matter where the membrane modules were located. Rare inherent holes could be observed on the outside ones, but there were some existing on inside surfaces, indicating more severe fouling. As for Module A, coccus covered by EPS were scattered obviously under high magnification. From A down to D, coccus became indistinct and foulants aggregated into a whole with more EPS covering, contributing to higher filtration resistance and consistent with the above distribution. In general, membrane fouling was caused by the combination of organic and biological foulants. Details are illustrated in Section 3.4.

### 3.4. Membrane Fouling Characterization

#### 3.4.1. Organic Foulants Identification

As shown in Figure S2, TOC concentration (15.20 mg/L) was the highest in the membrane chemical cleaning solution of the middle module (Module D). It decreased gradually from middle to top and bottom, displaying evidently more severe organic fouling of the middle part than the two sides, and the lightest fouling was in Module F.

**Figure 5:**
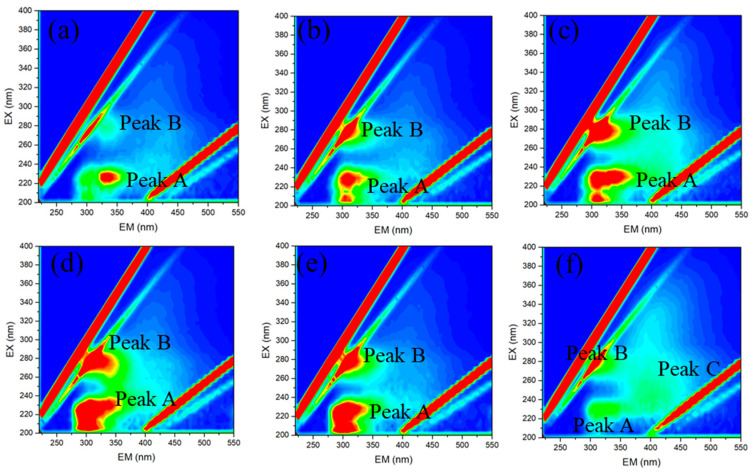
The 3D-EEM spectra of the membrane chemical cleaning soaks ((**a**) Module A; (**b**) Module B; (**c**) Module C; (**d**) Module D; (**e**) Module E; (**f**) Module F).

Fluorescence peak identification and fluorescence region integration (FRI) analyses of EEM spectra have been widely used in MBR studies to track the transformation of DOM and investigate the major contributors to membrane fouling [37]. In EEM spectra (Figure 5), two main peaks at excitation/emission wavelengths (Ex/Em) of 230/310 nm (Peak A) and 280/330 nm (Peak B) were observed, as a tyrosine-like protein and soluble microbial byproduct proved to induce *R_c_* by forming a dense gel layer [38]. These peaks suggested that the fluorogenic substrate was generally similar in the six modules. Besides these two, a different one at Ex/Em of 240/400 nm (Peak C) was observed as fulvic acid-like substance in the bottom membrane module (Module F). The relative intensity of Peak A and Peak B was higher in membrane D and E than in membrane modules A, B, C, and F. FRI results (Table 1) showed a similar spatial distribution trend. The ratios between fluorescence peak intensities were calculated, as shown in Table 1. The fluorescence index (FI) was close to 1.9 for the microbial sources of DOM in membrane foulants. The humification index (HIX), characterizing the maturity of DOM, was always far less than 4, suggesting in situ formation of dissolved organic foulants due to biological activities [39]. The biological source index (BIX) values of 0.8~1.0 indicate a strong autochthonous component of membrane foulants.

#### 3.4.2. Biological Foulants Identification

**Figure 6:**
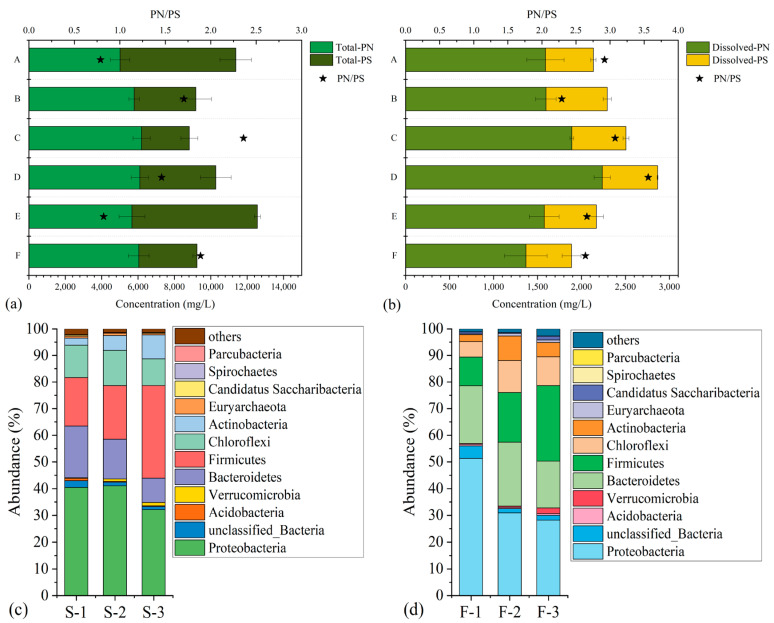
Contents and compositions of extracellular polymeric substances (EPS) in cake layer sludge (**a**,**b**); microbial community structures at phylum levels of bulk sludge (**c**) and foulants sludge (**d**).

**Figure 7:**
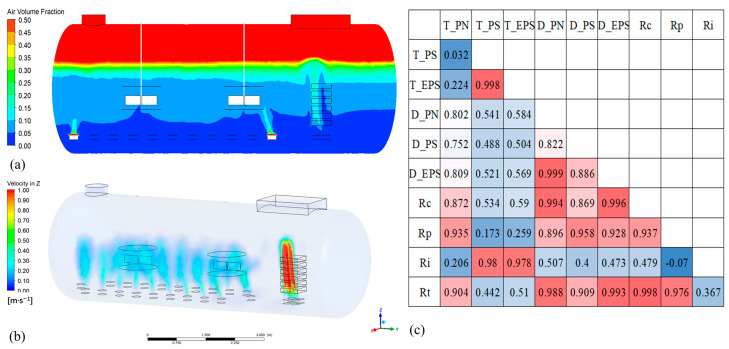
The simulated air volume fraction of ScMBR (**a**); the simulated velocity of ScMBR (**b**); Pearson correlation matrix presenting the correlations between fouling resistances in the contents of EPS (Blue and red colours represent negative and positive correlations, respectively.) (**c**).

Extracellular polymeric substances (EPS) are mainly secreted by microbes or lysed cells, containing polysaccharides (PS) and protein (PS) and playing important roles in cake layer formation. Therefore, a high concentration of EPS often induces serious membrane fouling. The spatial distributions of the total and the dissolved EPS of cake layer sludge from different membrane modules are illustrated in Figure 6a and Figure 6b, respectively. The spatial characteristics of dissolved EPS showed a significantly similar trend in total resistance (*R_t_*) and cake layer resistance (*R_c_*) (*p* < 0.05) (Figure 7c). They were higher in middle modules, but lower in both top and bottom modules. However, there was no similar pattern of total EPS (the sum of LB-EPS and TB-EPS) distribution at different positions. The results strongly support the findings of Niu et al. [40], who documented that LB-EPS, rather than TB-EPS, might be the major cause of membrane fouling. Wang et al. reported that PS and PN concentrations of SMP and EPS in a novel vibrating ceramic MBR (VMBR) were lower than in a conventional air-sparging MBR (ASMBR), and lower energy consumption and enhanced dewatering were observed in the ceramic VMBR [41]. Thus, it could be concluded that adopting vibration in ScMBR for SW treatment is helpful for enhancing the fouling mitigation produced by colloids and biopolymer clusters.

Furthermore, the PN/PS ratio was another critical factor, as shown in Figure 6. Lu et al. [42] demonstrated that EPS with a high content of proteins had strong glutinosity and high stickiness, causing a more evident cake layer formation because of the hydrophobic feature of protein. The dissolved PN/PS ratio ranged from 2.29 to 3.56 among six layers. The ratios of modules C (3.07) and D (3.56) were higher compared with the others, signifying higher fouling potential. The highest dissolved EPS concentration and PN/PS ratio of Module D tended to form negative fouling feedback, so it could be concluded that the fouling rate of Module D would be higher compared to the other modules. Thus, different cleaning measures and frequencies should be taken into account for membrane fouling control aimed at membrane modules located in different positions, as discussed in the following part.

The microbial community structure of cake layer sludge was investigated at the phylum level during three different operational phases. Proteobacteria, as a significant contributor to biological pollutant removal, was the dominant phylum, similarly to that in bulk sludge. In addition, Proteobacteria break down macromolecules (such as SMP) into small molecules [43], thereby mitigating fouling. Adversely, the suppression of Proteobacteria in the cake layer sludge (the abundance of Proteobacteria decreased from 51.31%, to 30.88%, to 28.19%) caused membrane fouling aggravation. Bacteroides, widely detected and reported to be involved in membrane fouling, ranged from 17.56% to 23.92%, higher than the abundance in bulk sludge (9.14~19.42%), and were namely enriched in the membrane surface. A similar observation was given by Liu et al. [43]. The reason might be that Bacteroidetes potentially release proteinaceous EPS, and fimbriae help them attach to the supporting material surface. Chloroflexi has been reported to contribute to carbohydrate biodegradation, but as filamentous bacteria, the growth of it may create a fixed layer and accelerate the cake layer formation [44]. The abundance of Chloroflexi increased from 5.81% to 10.83%, and it is partly responsible for the severe membrane fouling. Fimicutes are often detected as the dominant phylum in many kinds of anaerobic reactor, like those treating confectionery wastewater [27], methanolic wastewater [42], or cellulose wastewater [45]. The rapid increase in the abundance of Firmicutes (from 10.81% to 28.33%) may be related to the more anaerobic environment created by thick EPS layers.

### 3.5. Membrane Fouling Control

Taking the new membrane sheet as a reference, the membrane morphology of the stripped membrane after water cleaning and sequent chemical cleaning is shown in photos and SEM images (Figure S3). The membrane sheet from Module C was chosen as the typical example for analysis, because its *R_t_* was moderate and its ratio of *R_c_* and *R_p_* was equal to 1:1. Intuitively, the new membrane sheet was spotlessly white and there were many evenly distributed membrane pores. The surface looked smooth and no apparent substance was attached to it. The fouled membrane sheet stripped by water showed light brown bands. The membrane surface was covered with a mass of viscous substances. At higher magnification, the microbes can be faintly seen scattered in the cohesive material with a few small holes. The color of the membrane sheet stripped by chemicals was a uniform light yellow. Membrane pores reappeared in SEM images, though the surface was not as smooth as the new one. There was a thin layer of sticky material and several microbes spread across it, which could contribute to irreversible resistance (*R_i_*). *R_i_* took a very small proportion of *R_t_* for the membrane module of any position, consistent with the good recovery of membrane pores’ morphology, showing structural and pore stability [26].

The spatial distribution characteristics of membrane fouling could be related to the fluid dynamics in the ScMBR system, as shown in Figure 7a,b. The two-phase gas–liquid flow introduced by air bubbling is a technically effective strategy for fouling mitigation in submerged flat-sheet MBR systems [46], due to the high wall shear stress induced. CFD is an excellent tool in simulation, design, and optimization for complex hydrodynamics in membrane filtration processes [47]. In this study, gas flow, generated from spargers, split and developed into bubbles within the membrane channels, where bubbles interacted with the liquid flow and the membrane surface. At the same time, the bubbles coalesced or collapsed. The longitudinal extension of the membrane units is beneficial for bubbles’ development and uniform distribution in membrane channels. However, when extended to some height, the membrane sheets’ interaction would weaken the uniformity of bubble size. The closer the membrane assembly was to the aerator, the stronger the scouring effect was. Between E and B, increasing interaction time and frictional drag caused coalescence and breakup, and influenced bubble distribution. Around B, the unit extended, and the interaction frequency for the three phases of the gas–liquid–membrane surface increased as the height of the membrane module increased. Bubbles distributed uniformly, whether in the axial direction or the radial direction, which was helpful for scouring foulants on the membrane surface.

## 4. Conclusions

In this study, the ceramic membrane bioreactor showed a feasible and efficient performance in COD, TN, and NH_4_^+^ removal from high-strength swine wastewater. The dominant phylum in the whole microbial community was *Proteobacteria*, and the diversity of denitrifers was high, including *Pseudomonas*, *Comamonas*, *Thauera*, *Hyphomicrobium*, *Thermomonas*, *Paracoccus*, *Flavobacterium*, *Azoarcus*, *Thiobacillus*, and *Ralstonia*. The fouling degree of the ceramic membrane generally presented spatial difference. Membrane modules were heavily fouled in the middle position, and the total resistance reached 1.8 × 10^14^ m^−1^, but relatively less so at the top and the bottom. Tyrosine-like protein was the major contributor to organic fouling. A higher EPS and PN/PS ratio resulted in higher biological fouling in the form of *R_c_* and higher fouling potential of the middle module, related to the increasing abundance of Bacteroides, Chloroflexi, and Fimicutes in the cake layer microbial community. It is hoped that this study will support a developing strategy for using ScMBR technology with high COD and nitrogen removal efficiency in swine wastewater treatment. However, membrane fouling is still a major obstacle of ScMBRs. Further investigation will be carried out to reduce membrane fouling, ideally with a long-term effect, using dispersed high-flux gas scouring and in situ chemical cleaning.

## Figures and Tables

**Table 1:** List of FI, HIX, BIX values and results of FRI.

Membrane Module	Indicators Based on Peak Intensity Ratios			FRI Results of Five Regions (10^6^)					FRI Proportion (%)				
	FI	HIX	BIX	I	II	III	IV	V	I	II	III	IV	V
A	1.87	0.50	0.82	10.1	15.2	5.7	5.9	3.2	0.25	0.38	0.14	0.15	0.08
B	1.92	0.39	0.85	15.1	13.9	5.8	8.6	3.0	0.33	0.30	0.12	0.19	0.06
C	2.15	0.40	0.94	17.4	24.0	8.9	14.4	5.0	0.25	0.34	0.13	0.21	0.07
D	2.01	0.27	0.89	25.3	24.0	7.2	15.0	3.7	0.34	0.32	0.10	0.20	0.05
E	2.12	0.31	0.99	22.9	16.1	5.8	10.4	2.8	0.39	0.28	0.10	0.18	0.05
F	1.83	0.53	1.04	7.1	13.2	7.8	9.6	5.7	0.16	0.31	0.18	0.22	0.13

## Data Availability

Data are contained within the article or the Supplementary Materials.

## Supplementary Materials

The following supporting information can be downloaded at: https://www.mdpi.com/article/10.3390/membranes14060142/s1, Figure S1: Microbial community of bulk sludge at genus level; Figure S2: TOC concentrations of membrane cleaning soaks of different membrane modules; Figure S3: Real images and SEM images of membrane sheet of Module C at different magnifications (N—New; W—Water Cleaning; C—Chemical Cleaning).
