# Supplementary material for: The Performance and Spatial Distribution of Membrane Fouling in a Sequencing Batch Ceramic Membrane Bioreactor: A Pilot Study for Swine Wastewater Treatment
Source: Membranes (Basel). 2024 Jun 18;14(6):142. doi: 10.3390/membranes14060142 (PMC11206136; doi:10.3390/membranes14060142)
Supplement: Supplementary file 1 [file membranes-14-00142-s001.zip › membranes-3012033-supplementary.pdf]

Supplemental materials

Performance and spatial distribution of membrane fouling in a sequencing-batch ceramic membrane bioreactor: A pilot study for swine wastewater treatment

Wenhui Yue[1,2,3], Yanlin Chen[1,2], Qianwen Sui[1,2], Libing Zheng[1,2], Tharindu Ritigala[1,2], Yuansong Wei[1,2,3,*]

1. State Key Joint Laboratory of Environment Simulation and Pollution Control, Research Center for Eco-Environmental Sciences, Chinese Academy of Sciences, Beijing 100085, China; wenhui_yue@163.com(W.Y.); chen_yanlin@ct.com.cn(Y.C.); qwsui@rcees.ac.cn(Q.S.); lbzheng@rcees.ac.cn(L.Z.); tharinduritigala@live.com(T.R.)
2. Laboratory of Water Pollution Control Technology, Research Center for Eco-Environmental Sciences, Chinese Academy of Sciences, Beijing 100085, China
3. University of Chinese Academy of Sciences, Beijing 100049, China

* Correspondence: yswei@rcees.ac.com

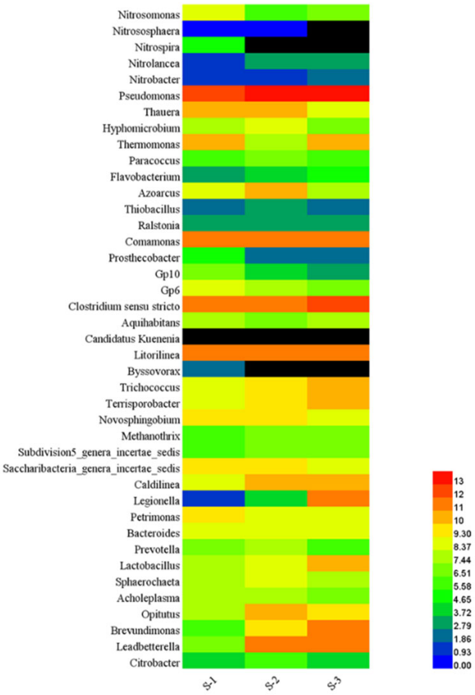

Figure S1. Microbial community of bulk sludge at genus level.

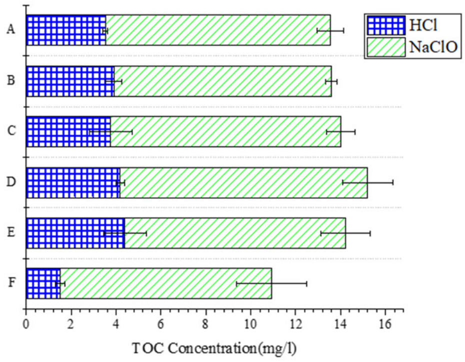

Figure S2. TOC concentration of membrane cleaning soak of different membrane modules.

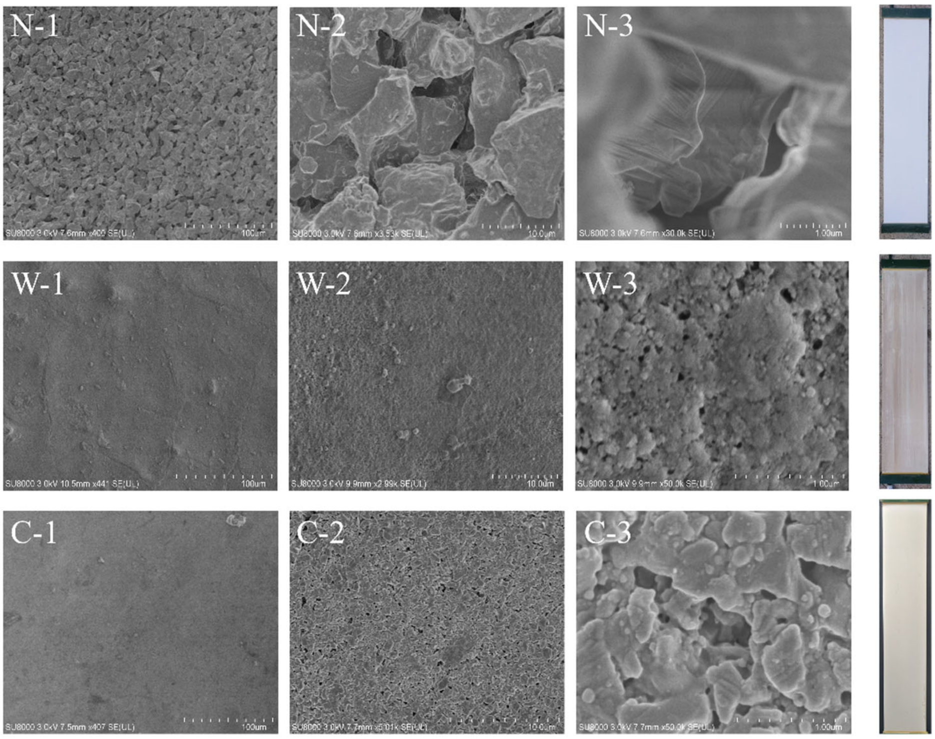

Figure S3. Real images and SEM images of membrane sheet of module C at different magnifications (N-New; W-Water Cleaning; C-Chemical Cleaning).
